# Editorial: Drug resistance in lung cancer chemotherapy and personalized chemotherapy

**DOI:** 10.3389/fcell.2022.971477

**Published:** 2022-08-16

**Authors:** Lianbo Li

**Affiliations:** Departments of Radiation Oncology, The University of Texas Southwestern Medical Center at Dallas, Dallas, TX, United States

**Keywords:** lung cancer, chemotherapy, drug resistance, chemoresistance, personalized chemotherapy, precision chemotherapy

The Research Topic “*Drug Resistance in Lung Cancer Chemotherapy and Personalized Chemotherapy*” was launched by *Frontiers in Cell and Developmental Biology: Cancer Cell Biology* and *Frontiers in Oncology: Molecular and Cellular Oncology* in July 2021 to serve as an open-access venue for outstanding research and reviews in the field. Due to the importance of lung cancer research, and the challenges that remain for chemotherapy, it attracts dozens of high-quality original research manuscripts. The published papers related to our Research Topic cover a broad swath in the area of non-small cell lung cancer (NSCLC) and small cell lung cancer (SCLC), from kinase mutation related drug resistance, to clinical application of combination therapy, to case studies of pemetrexed treatment. This editorial provides a broad overview of research on the topic, highlighting significant contributions to the field. Due to a limit on the number of words allowed in the editorial, many interesting insights and results are not discussed, but deserve the same attention. We highlight the following publications to exhibit the breadth and depth of these scholarly contributions.

Lung cancer is one of the most common causes of cancer-related death, and non-small cell lung cancer (NSCLC) accounts for more than 80% of all lung cancers. Most patients are diagnosed at advanced stages as early-stage symptoms cannot be clearly diagnosed. Both drug-eluting bead bronchial arterial chemoembolization (DEB-BACE) and anlobinib (an oral multi-targeted tyrosine kinase inhibitor) have proved to be effective and relatively safe in treating advanced NSCLC patients. Liu et al. started from the hypothesis that DEB-BACE and anlotinib combination therapy might provide promising outcomes in treating advanced NSCLC patients. Using data directly extracted from clinical patients, the author evaluated anticancer activity and concluded that such a combination had promising efficacy and tolerable toxicity in advanced NSCLC patients. Although several similar studies have been reported previously, this study is still valuable given the direct clinical investigation of novel combination therapy.

Ground glass opacity (GGO) refers to the hazy gray areas that can show up in CT scans or X-rays of the lungs, and is widely considered a prognostic factor for lung adenocarcinoma (LUAD) patients. In the article by Zhai et al. the authors used data from 501 IB–IIA LUAD patients at Sun Yat-sen University Cancer Center from January 2008 to June 2018 to evaluate GGO in predicting stage IB–IIA lung adenocarcinoma patient survival rates. By integrating GGO into their nomogram for predicting disease-free survival (DFS) and overall survival (OS), they found that the factor consolidation-to-tumor ratio (CTR) < 0.75 is associated with better DFS in patients with stage IB–IIA LUAD. They also evaluated the impact of adjuvant chemotherapy (ACT) on pathological stage IB–IIA LUAD, and developed prognostic nomograms for individual OS and DFS, which may assist in selecting patients that might benefit from ACT. Overall, this study broadens our current knowledge of GGO in LUAD and provides new insights for clinical application.

Anaplastic lymphoma kinase (ALK) is a known therapeutic target in non-small cell lung cancer (NSCLC) but drug resistance prevents its further application. To overcome the challenge of clinically acquired drug mutations for targeted therapies and personalized medicines, Liang et al. used computer-aided drug development methods, such as molecular docking and multiple molecular dynamics simulations, to elucidate the mechanism of resistance to ALK inhibitors. With energetic and structural analyses, they first proposed a structural model of an ALK−gilteritinib complex, and found that double mutations I1171N/F1174I reduce the dynamic states of the ALK kinases. Two conserved residues, Glu1197 and Met1199, are responsible for hydrogen bonding interactions critical for drug resistance. Overall, this study broadens our current understanding of drug resistance in ALK kinase and provides new insights into the design of next-generation anti-tumor drugs.

Pemetrexed is an established therapy for patients with advanced non-squamous non-small cell lung cancer (NSCLC). However, the outcomes of pemetrexed when extended to NSCLC with squamous harboring driver oncogenes remain unclear. In the case report written by Patil et al. the authors identified two NSCLC patients harboring an ALK and ROS1 gene arrangement with potential adenosquamous carcinoma. Unlike other squamous NSCLC patients who rarely benefit from pemetrexed treatment, they responded to therapy, with increased survival rate benefits clinically evident. The proposed pemetrexed treatment for squamous NSCLC patients is novel and these real-world patient responses indicate that similar treatment could be beneficial for squamous patients with ALK and ROS1 gene arrangements in the future.

Platinum-based chemotherapy is the first-line treatment for small cell lung cancer (SCLC). However, this treatment is only effective for some patients. Most patients develop drug resistance, which leads to disease progression. A significant body of research has shown that gene mutations affect drug resistance in various cancer types. It is not clear what genetic background is responsible for sensitivity to platinum drugs in SCLC. Yi et al. analyzed whole-exome sequencing (WES) and clinical data from two cohorts to find gene mutations related to platinum-based chemotherapy sensitivity. Their results showed that CAMSAP1 mutation was associated with a better overall survival in both cohorts, suggesting that CAMSAP1 mutation can increase platinum sensitivity and works as a suitable biomarker to guide platinum-based chemotherapy for SCLC. They also performed GSVA and GSEA analyses to investigate possible pathways and mechanisms and to search for potential therapeutic drugs. This important work has significance in both basic and clinical research.

